# Degraded tropical rain forests possess valuable carbon storage opportunities in a complex, forested landscape

**DOI:** 10.1038/srep30012

**Published:** 2016-07-20

**Authors:** Mohammed Alamgir, Mason J. Campbell, Stephen M. Turton, Petina L. Pert, Will Edwards, William F. Laurance

**Affiliations:** 1 Centre for Tropical Environmental and Sustainability Science (TESS) and College of Science and Engineering, James Cook University, Cairns, Queensland 4870, Australia; 2 Central Queensland University, Cairns, Queensland 4870, Australia; 3 CSIRO Land and Water, P.O. Box 12139, Earlville, Queensland 4870, Australia

## Abstract

Tropical forests are major contributors to the terrestrial global carbon pool, but this pool is being reduced via deforestation and forest degradation. Relatively few studies have assessed carbon storage in degraded tropical forests. We sampled 37,000 m^2^ of intact rainforest, degraded rainforest and sclerophyll forest across the greater Wet Tropics bioregion of northeast Australia. We compared aboveground biomass and carbon storage of the three forest types, and the effects of forest structural attributes and environmental factors that influence carbon storage. Some degraded forests were found to store much less aboveground carbon than intact rainforests, whereas others sites had similar carbon storage to primary forest. Sclerophyll forests had lower carbon storage, comparable to the most heavily degraded rainforests. Our findings indicate that under certain situations, degraded forest may store as much carbon as intact rainforests. Strategic rehabilitation of degraded forests could enhance regional carbon storage and have positive benefits for tropical biodiversity.

Although tropical forests cover only ~6% of the global land surface, they are the largest single repository of above-ground biomass carbon (ABC) stores^1^,^2^,^3^, containing ~195 petagrams of carbon (PgC)^4^,^5^. In addition to their total ABC storage, tropical forests are also net carbon sinks^5^,^6^,^7^. As a consequence of their significant carbon storage and sink capacity, tropical forests play a critical role in climate change mitigation^8^,^9^. However, despite this valuable carbon storage and potential climate change mitigation capacity, tropical forests experience high levels of annual deforestation^10^,^11^, which has been estimated to have resulted in an annual, global ABC loss of 0.26 PgCYr^−1^ over the period 1993–2012^4^. Moreover, deforestation of tropical forests is the second greatest contributor of green-house gas emissions to the atmosphere after the burning of fossil fuels^12^,^13^.

In addition to deforestation, much of the remaining tropical forested area experiences various forms of degradation with the area of degraded tropical forests now estimated to exceed 500 million hectares^14^. Moreover, regenerating forests are estimated to exceed primary forests as the predominant form of tropical forest cover worldwide^15^. Degraded tropical forests store less ABC than primary forests^16^,^17^,^18^ and as such forest degradation results in increased atmospheric CO_2_ emissions^16^,^17^,^19^. Alternatively, however, forest regrowth within degraded forests may remove large amounts of carbon from the atmosphere^5^. As such, it is becoming crucial to determine the impact of forest degradation and regeneration on net CO_2_ emissions and overall forest carbon storage capacity^16^,^17^,^20^,^21^,^22^.

One problem faced when determining the carbon storage capacity of degraded forests (and thus their net CO_2_ emissions) within complex tropical forest regions is that landscape-scale factors often determine the impact of forest degradation on carbon storage. These factors include: variability in the availability of constraining environmental resources^23^,^24^, differences in vegetation composition^25^,^26^, forest structural variation^27^,^28^, and the applied forest management regime^8^,^29^. As a whole, it is still unclear what impact each of these factors has on the dynamics of carbon storage in complex tropical forested landscapes^23^,^30^. As such, if the development of optimal management strategies to enhance landscape-scale carbon storage in complex tropical forested landscapes is to occur, determining the impact of individual landscape-scale factors on the carbon storage capacity of degraded forests is of utmost importance^20^,^31^.

The Wet Tropics bioregion of northeast Australia is a complex and contested (for land use) landscape^32^ which is primarily composed of one of the oldest rainforests on earth^33^. This region has been described as the second most irreplaceable natural world heritage area^34^, and the sixth most irreplaceable protected area on the planet^35^. The significant biodiversity values of the Wet Tropics bioregion are well documented^36^,^37^,^38^. However, the carbon storage values of the component vegetation types within the Wet Tropics bioregion is yet to be determined – precluding carbon storage within restoration plantings of the area^39^.

Here we evaluate, the biomass and carbon storage of intact closed-canopy forests (hereafter termed rainforest), degraded closed-canopy forests (hereafter termed degraded forest) and sclerophyll forests within a complex and heterogeneous landscape of the Wet Tropics bioregion. In addition, we examine the influence of forest structural features (e.g. tree size) and disturbance upon forest biomass and carbon storage. We also compare the impact of rainfall and elevational gradients upon the carbon storage of these vegetation types. Finally, we discuss mechanisms whereby appropriate site selection and management of degraded forests, may allow for potential policy interventions which enhance carbon storage in the tropical forests of this landscape and additionally aid biodiversity conservation.

## Results

### Structural variation across the forest types

**Figure 1:**
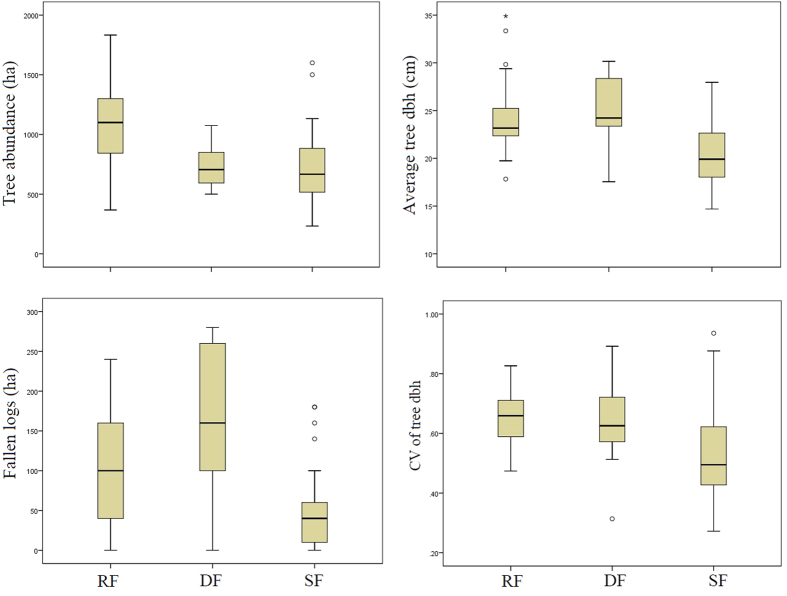
The average number of trees (≥10 cm dbh) per ha, average tree diameter at breast height (cm), average number of fallen logs (≥10 cm diameter) and average coefficient of variance (CV) of tree diameter at breast height (cm) in the examined plots of rainforest (RF) (n = 29), degraded forest (DF) (n = 13) and sclerophyll forest (SF) (n = 32) within the Wet Tropics bioregion of northeast Australia.

We counted and measured a total of 1,438 trees in rainforests, 1193 trees in degraded forests and 693 trees in the sclerophyll forests. There was a significant difference in the number of trees ≥10 cm dbh among the examined forest types (χ^2^ = 18.269, df = 2, p_2-tailed_ = <0.001), with a pairwise post hoc comparison, showing that rainforests (RF) had significantly more trees than degraded forest (DF) and sclerophyll forests (SF) (RF-DF p_2-tailed_ = 0.013; RF- SF p_2-tailed_ = <0.001) (Fig. 1). However, there was no significant difference in the number of trees between degraded forests and sclerophyll forests (p_2-tailed_ = 1.00) (Fig. 1). The average tree dbh (cm) was significantly different among the three forest types (χ^2^ = 16.295, df = 2, p_2-tailed_ = <0.001), with rainforests and degraded forests possessing significantly larger trees than sclerophyll forests (RF- SF p_2-tailed_ = 0.002; DF- SF p_2-tailed_ = 0.004) (Fig. 1). Analogously, the number of fallen logs (≥10 cm diameter) per ha was significantly different among the forest types (χ^2^ = 15.406, df = 2, p_2-tailed_ = <0.001), with the post hoc pairwise comparison again finding that rainforests and degraded forests possessed more fallen logs than sclerophyll forests (RF- SF p_2-tailed_ = 0.006; DF- SF p_2-tailed_ = 0.002) (Fig. 1). Finally, the co-efficient of variance (CV) of tree dbh was also significantly different among the forest types (χ^2^ = 13.689, df = 2, p_2-tailed_ = 0.001), which was again driven by the significantly larger values of CV of tree dbh in the rainforest and degraded forests compared to the sclerophyll forest (RF- SF p_2-tailed_ = <0.001; DF- SF p_2-tailed_ = 0.046) (Fig. 1).

### Above ground biomass and carbon stock

**Figure 2:**
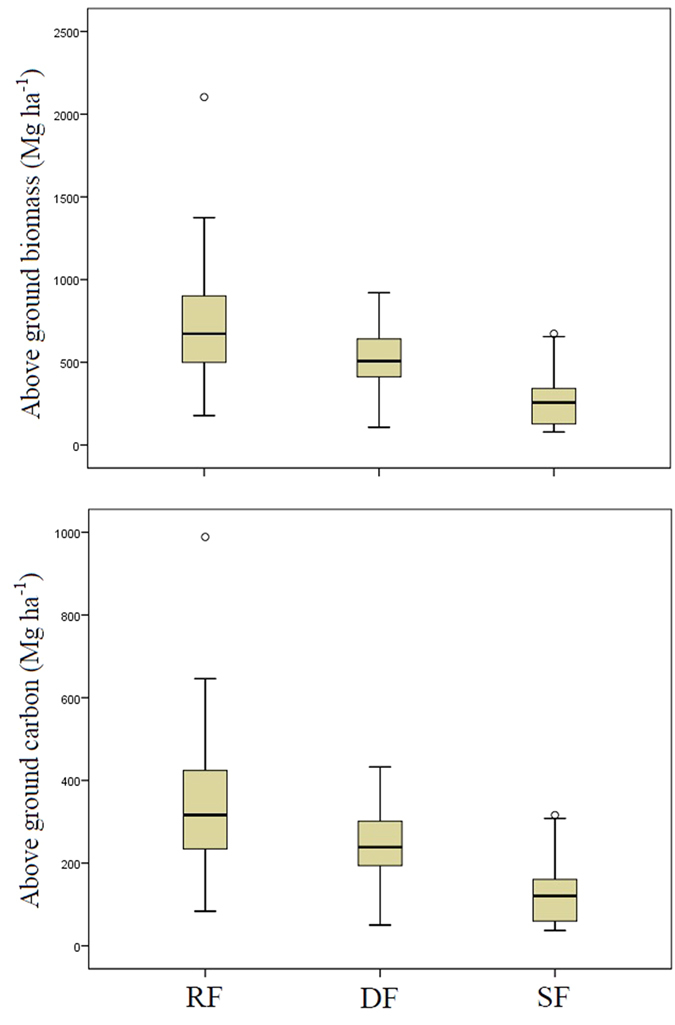
The above ground biomass (Mg ha^−1^) and above ground carbon (Mg ha^−1^) stored within the examined rainforest (RF) (n = 29), degraded forest (DF) (n = 13) and sclerophyll forest (SF) (n = 32) vegetation types of the Wet Tropics bioregion of northeast Australia.

There was a significant difference among the amount of above ground biomass (Mg ha^−1^) and above ground carbon (Mg ha^−1^) stored within the examined rainforests, degraded forests and sclerophyll forests (χ^2^ = 33.064, df = 2, p_2-tailed_ = <0.001 in each case). Using a pairwise post hoc comparison, we found that there was significantly more above ground biomass (Mg ha^−1^) and above ground carbon stored (Mg ha^−1^) within the rainforests and the degraded forests than within the sclerophyll forest (p_2-tailed_ = <0.001 in each case) (RF- SF p_2-tailed_ = <0.001; DF- SF p_2-tailed_ = 0.009). However, there was no significant difference in the amount of above ground biomass and above ground carbon stored in the rainforests and degraded forests (RF- DF p_2-tailed_ = 0.442) (Fig. 2).

### Variation and distribution in carbon storage of the examined vegetation types

**Figure 3:**
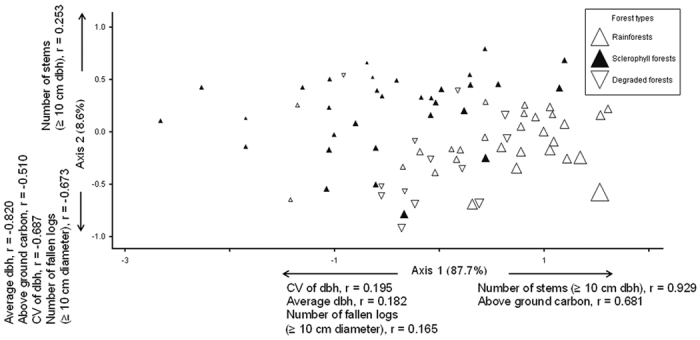
Nonmetric Multidimensional Scaling (NMS) ordination showing the variability of above ground carbon storage in relation to other forest attributes of plots located in rainforests (n = 29), degraded forests (n = 13), and sclerophyll forests (n = 32) of the Wet Tropics bioregion, of northeast Australia. The size of the dots indicates relative quantity of above ground carbon storage. The x-axis (axis 1) represents 87.7% (r^2^) and the y-axis (axis 2) 8.6% (r^2^) of the described variation.

The Nonmetric Multidimensional Scaling (NMS) ordination of above ground carbon storage of plots in variable space explained 96.3% cumulative variation in the examined data, where the x-axis (axis 1) represented 87.7% and y axis (axis 2) 8.6% of the variation (Fig. 3). The x-axis showed a strong correlation with the number of trees and above ground carbon storage variables (r = 0.929, and 0.681 respectively), whilst the y axis showed a strong correlation with the average tree dbh and above ground carbon storage variables (r = −0.820, and −0.510 respectively) (Fig. 3).

Most of the rainforest plots had higher levels of above ground carbon storage than those of sclerophyll forests (Fig. 3), whilst the above ground carbon storage in the degraded forest plots varied from high levels similar to those of rainforests through to low levels similar to plots within sclerophyll forests (Fig. 3). As such, rainforests plots and sclerophyll forests plots occupied somewhat distinct ordination spaces whilst those of the degraded forest plots intergraded between the two; though they were, in general, more similar to those of the rainforest plots (Fig. 3).

### Factors determining carbon stock

**Figure 4:**
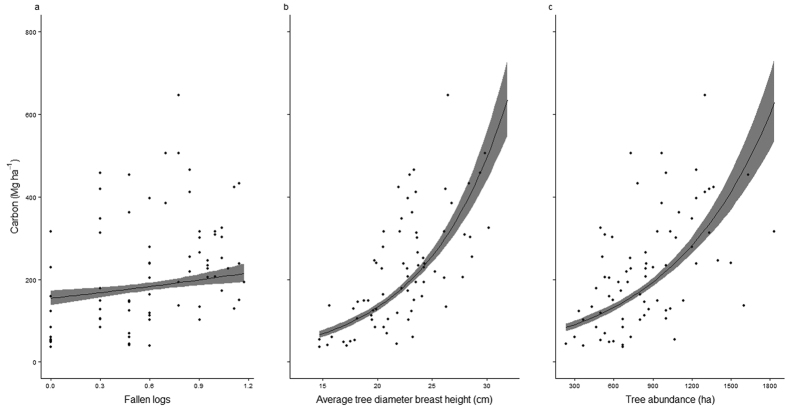
The significant relationship between (**a**) fallen logs (≥10 cm diameter) ha^−1^ (log transformed), (**b**) average tree diameter breast height (cm) and (**c**) tree abundance and carbon storage (Mg ha^−1^) of the examined plots, within the Wet Tropics bioregion northeast Australia. Filled circles represent the plot (50*10 m) values. The trend line was constructed using a binomial GLM with logit link function and shaded areas represent the 95% confidence intervals.

The backwards, stepwise, negative binomial generalized linear model (GLM) process identified three significant explanatory variables for determining the carbon storage of plots (R^2^ = 0.888; null deviance-residual deviance/null deviance): the number of fallen logs per plot, average tree diameter breast height per plot and the tree abundance per plot (Fig. 4a–c and Table 1). All of these explanatory variables displayed a positive correlation with the carbon storage (Mg ha^−1^) of plots (Fig. 4a–c and Table 1).

## Discussion

We found that rainforests within the Wet Tropics bioregion store the highest levels of above ground biomass carbon (ABC) of the three examined vegetation types and when these forests experience degradation their storage capacity is reduced (Figs 2 and 3). However, the reduction in ABC storage values of plots in degraded forests compared with those of rainforest was not significant (Fig. 2) although degraded forest plots did display considerably more variation in ABC storage (Fig. 3). We suggest that it is likely that the examined degraded forests are in an advanced stage of regeneration given the lack of significant difference between their average tree size (dbh) and that of the rainforest. Moreover, the examined degraded forests on a global spectrum still store proportionately high values of ABC (241.04 ± 27.09 Mg ha^−1^) when compared with other degraded tropical forests. For example, our reported values of ABC for the examined degraded forests (Fig. 2) are higher than those of Ioki, *et al*.^20^ who estimated 52.18–229.11 Mg ha^−1^, and 136.00–382.59 Mg ha^−1^ ABC for the highly degraded and moderately degraded tropical rain forests of northern Borneo, and for those of Usuga, *et al*.^27^ who reported 99.6 Mg ha^−1^ and 85.7 Mg ha^−1^ ABC storage from Tropical Pine and Teak forest plantations in Colombia. Nonetheless, our observation of a non-significant decline in the ABC storage capacity of degraded forests compared to non-degraded rainforests is supported by findings reported elsewhere from within the tropics^16^,^17^,^18^,^40^.

Much of the decline in ABC storage within the degraded forests we examined appears to be due to increased disturbance. For instance, degraded forests when compared with non-degraded rainforests were found to display less trees (Fig. 1) and possess a higher number of fallen logs (Fig. 1). Furthermore, many of the degraded forests we examined were fragmented, a process which is known to result in higher levels of forest disturbance and tree loss through an increased susceptibility to wind damage^41^ and an altered microclimate on forest edges^21^,^42^. Moreover, both wind damage and microclimatic alterations within forests degraded by fragmentation are known to result in the disproportionate loss of large trees, especially on forest edges^18^,^43^,^44^. This loss of large trees can significantly alter the carbon storage capacity of degraded forests as large trees are known to drive tropical forest ABC storage^45^. In addition, forest fragmentation is known to increase wind damage susceptibility which may be particularly pertinent to lowland forests of this geographic region as they are exposed to regular cyclonic impacts, with the greater Wet Tropics bioregion experiencing 45 recorded east-to-west moving tropical cyclone impacts over the period 1858–2011^46^,^47^. Therefore, strategies that minimize the disturbance of degraded forests and especially forest edges, might allow for enhanced ABC storage through successional recovery of the tree community and re-instatement of resilience to natural disturbances, particularly within larger fragments of forest. For instance, employing wind disturbance mitigation strategies such as wind-buffer plantings along the forest edges^48^ of the degraded forests of the region could substantially assist in decreasing forest disturbance^41^ and thus increase the ABC storage of these forests.

Sclerophyll forests within the study region had the smallest average tree size (dbh) (Fig. 1) and stored the least ABC of the three examined vegetation types (Fig. 2). The small average size (dbh) of trees within the sclerophyll forests compared to the other vegetation types is unsurprising given the less productive environmental envelope this vegetation type occupies (i.e. lower rainfall) and the fact that sclerophyll forests are pyrophytic and as such recruitment events are often determined by fire events and the time intervals between these^49^. Previous work^50^ has suggested that one of the main determinants of the distribution of the rainforest and sclerophyllous vegetation types within the examined region, may be fire, especially given the pyrophobic nature of the rainforest vegetation^49^,^50^,^51^. Therefore, management practices that aim to optimize ABC storage within the Wet Tropics bioregion whilst supporting the different species assemblages housed by rainforest and sclerophyll forests should, where practicable and appropriate, focus on the exclusion of fire from the rainforest component of the landscape to allow for the successional repair of the degraded rainforest vegetation type.

In addition to the exclusion of fire from the degraded forests, assisted restoration of these forests within the Wet Tropics bioregion may be an effective management strategy to allow for significant net ABC storage gains. This is suggested as the degraded forests within the region vary in their ABC storage capacity from values at a low end similar to sclerophyllous vegetation through to those of comparable non-degraded rainforests (Fig. 3). In particular, restoration of localized factors which support the retention of large trees and increase tree abundance would significant increase ABC storage across all forest types and within the degraded forest in particular (Figs 3 and 4, Table 1; Slik, *et al*.^45^). Although an increase in the number of fallen logs (as proxy for disturbance) was also found to increase the ABC storage capacity of the examined forests (Fig. 4); it is highly likely (given previous research on forest disturbance^52^,^53^) that increased disturbance within these forests would result in an asymptote and eventual negative relationship occurring between the number of fallen logs and ABC storage. As such, utilizing intermediate disturbance to attain increase ABC storage of forests in the studied region would be problematic and impractical.

Finally, the use of land within the Wet Tropics bioregion is highly contested^32^. Consequently, within this landscape, multi-value land usage strategies may maximize the likelihood of degraded forest retention. For instance, as well as their significant ABC storage values (Fig. 2), tropical rainforests are known to house the zenith of terrestrial biodiversity^54^. Additionally, remnant, fragmented and degraded forests can provide an important biodiversity repository for many complex tropical landscapes^21^,^55^,^56^. Consequently, degraded forest management within the Wet Tropics bioregion provides considerable opportunities for integrating ABC storage values with biodiversity conservation. In particular, the uplands of the Wet Tropics bioregion, as well as providing an area of low disturbance and thus optimal locations for ABC storage in both rainforest and degraded forests (Fig. 3), are also a known “hotspot” for endemism and diversity of numerous biota^57^,^58^,^59^ many of which are under threat^38^,^60^. Moreover, recent studies of forest restoration within this region suggest that secondary and degraded forests restoration may be passively enhanced through selection of sites in close proximity to primary forest^61^; although if maximal biodiversity outcomes are to be gained specific species may need to be actively restored^62^. Finally, rainforest restoration should not occur at the expense of other distinct, remnant vegetation types such as sclerophyll forests as a net biodiversity loss could occur due to the different species assemblages they support^32^.

## Conclusion

To maximise ABC storage within the complex landscape of the Wet Tropics bioregion, it is optimal to conserve primary rainforests at sites that experience low levels of disturbance. Additionally, although degraded forests do not store as much ABC as non-degraded rainforest they play a considerable supplementary role in ABC storage and given appropriate management (i.e. disturbance minimization through fire exclusion and edge buffer plantings) and sufficient recovery, they can store as much ABC as non-degraded rainforest. Any additional ABC storage provided by degraded forests will come through the accumulation of additional carbon from atmosphere and thereby contribute to climate change mitigation. In addition, if degraded forests in close proximity to primary forests can be restored and supplementarily seeded with selected tree species they may also provide additional and considerable biodiversity conservation capacity.

## Methods

### The study area

**Figure 5:**
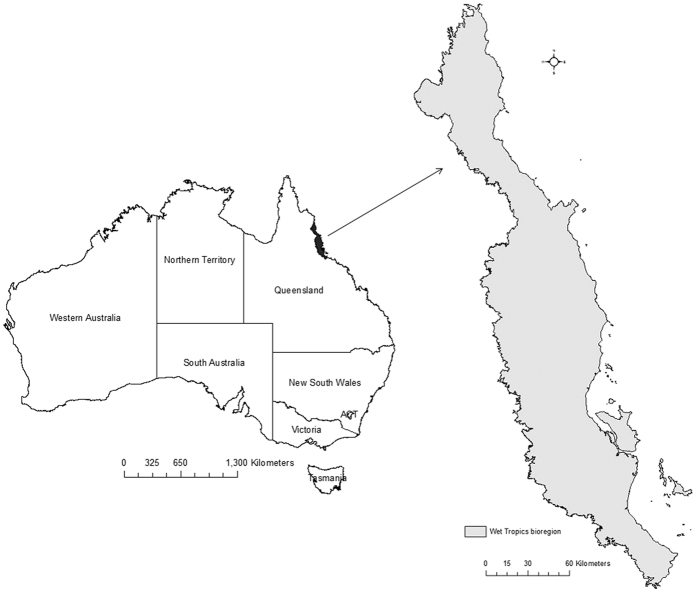
The study area- Wet Tropics bioregion, Queensland Australia. The maps were created using Esri ArcMap 10.2. (http://www.arcgis.com).

Our study was conducted in the Wet Tropics bioregion, northeast Australia (Fig. 5). The total area of the bioregion is ca. two million ha^63^, most of which experiences a seasonally wet tropical climate. The total mean annual rainfall ranges from 1200 mm to 4000 mm (although the highest mountain peaks may receive 8000 mm yr^−1^) and the mean annual temperature ranges from 17 °C to 31 °C^63^. The elevation of the bioregion ranges from a few meters above mean sea level (msl) to ~1000 m although the highest peak within the region is 1622 m. The heterogeneous and complex landscape of the region is dominated by contiguous rainforests and sclerophyll forests with environmentally defined boundaries^50^. The structure, composition and distribution of rainforests and sclerophyll forests in the Wet Tropics bioregion are largely determined by rainfall, elevation and soil types^64^,^65^.

### Attributes of studied forest types

The rainforests of the Wet Tropics bioregion are the largest remaining rainforests in Australia^66^, contain globally recognized biodiversity^35^ and provide valuable ecosystem services^67^. The rainforests within the study area are the remnants (and regrowth) of a formerly much larger rainforest expanse that covered large portions of the greater Wet Tropics bioregion. Significant deforestation of the area began in approximately 1880 and proceeded rapidly during the next 5 decades^68^. Moreover, most of the remaining rainforests of this region have been selectively logged since 1880 for valuable hardwood timber species such as Red Cedar (*Toona ciliata*)^69^. However, all logging of approximately, 45% (894,420 ha) (mainly rainforest) of the Wet Tropics bioregion ceased with its inscription on the World Heritage list in 1988 as a property that fulfilled all four natural criteria for listing^37^,^63^. The examined rainforests within the study area can generally be described as complex mesophyll to notophyll vine forest (regional ecosystem 7.8.2 and 7.8.4) with drier areas transitioning into complex semi-evergreen notophyll vine forest (regional ecosystem 7.8.3)^65^,^70^. Within the complex mesophyll vine forest, multiple continuous canopies may be present with the upper canopy averaging a height of 20–40 m^65^. Deciduous tree species are rare, however woody lianas, epiphytes and ferns are quite common resulting in a complex forest structure^65^,^70^.The rainforests of this region are biologically diverse and dominated by trees from Lauraceae, Moraceae, Myrtaceae, Rutaceae and Sapindaceae families^71^. The sclerophyll forests of this region are relatively open, with a grassy understory and the canopy is dominated by trees of the following genera: *Eucalyptus, Corymbia, Melaleuca, Acacia, Allocasuarina, Casuarina, Lophostemon* and *Syncarpia*^65^. We defined degraded forest as rainforest that was in the process of recovering after extensive disturbance (e.g. logging or clearing) or were fragmented.

### Sampling regime and data collection

We sampled a total of 74 plots: 29 rainforest, 32 sclerophyll forest and 13 degraded forest plots. In each forest types, sampling points were predetermined using ESRI ArcGIS 10.2 prior to field data collection to a) avoid creeks and water bodies; and b) minimize edge effects by maintaining at least 20 m distance between our plots and other land uses. Forest types in the Wet Tropics bioregion are distributed across a wide range of environmental gradients^65^. Therefore, we sampled sites across an: elevation gradient ranging from 12 meters above mean sea level (msl) to more than 1000 m; across a mean annual rainfall gradient ranging from less than 1000 mm to more than 3500 mm mean annual rainfall; and across a wide range of soils including: alluvium, genesis, fine sedimentary, laterite and andesite. Mean annual rainfall data was determined using long-term records for the region provided by the Wet Tropics Management Authority, Queensland, Australia^72^. Finally, all the sites were georeferenced and general environmental and landscape features such as slope and elevation recorded.

The size of each sampling plot was 0.05 ha (50 m * 10 m transect). We adopted 0.05 ha as our plot size as this has been previously found suitable to adequately sample widely spaced large trees, along with the relatively more smaller trees^39^,^73^. Moreover, for our data collection we used a transect method which has previously been determined suitable for estimating high densities of trees in rainforests^39^ and sclerophyll forests^74^. We measured the diameter at breast height (dbh) of all trees ≥10 cm within our plots to the nearest millimeter. In each of the plots we also counted the number of fallen logs ≥10 cm diameter on the forest floor (≤1 m above ground level).

### Estimation of above ground biomass carbon

Preece, *et al*.^39^ compared the accuracy of biomass estimation methods for forests within the Wet Tropics bioregion and concluded that the Chave, *et al*.^75^ allometric provided the best and most reliable estimate for the region. As such, we estimated above ground biomass (AGB) following Chave’s allometric equation^75^. Moreover, the Chave, *et al*.^75^ allometric was especially developed for tropical forests using data from 27 sites across the tropics, where 2,410 trees ≥5 cm dbh (with maximum 156 cm) have been directly harvested^75^, and subsequently found suitable for small to large diameter range trees^39^. To convert AGB into biomass carbon storage we used a conversion factor of 0.47 which is the recommended value from the Intergovernmental Panel for Climate Change for tropical forests^76^. Like other regions in the world, wood density in the trees of Wet Tropics forests vary widely from species to species and across different forest types^77^ and due to the diversity of the examined tropical forest many species specific densities are still as yet unknown. To compensate for this uncertainty, in this study wood density estimates were calculated using the Australian Governments Department of Climate Change and Energy Efficiency reported default value for Australian tropical forests of 0.5 g cm^−3^ (500 kgm^−3^)^78^. Whilst this approach may underestimate or overestimate the above ground carbon storage in some plots it utilizes the best current knowledge on tropical Australian rainforest tree species average wood density whilst still remaining practicable. Moreover, the present study still provides a clear comparative analysis of above ground carbon storage across the examined forest types, though of course, were it available, using species level wood density would produce more precise results. Consequently, AGB was calculated using equation (1):


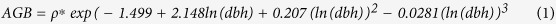


where AGB is measured in kg, dbh is measured in cm, and *ρ* is wood density measured in g cm^−3^.

Above ground biomass estimates were then converted to carbon estimates using equation (2):


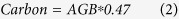


### Statistical analyses

All statistical analyses were conducted in IBM SPSS 20, PCORD 6 and Program R^79^. We used independent Kruskal-Wallis tests (2-tailed, α = 0.05) to compare above ground biomass, above ground carbon storage and forest structural attributes (number of stems (≥10 cm dbh), average tree dbh, fallen logs (≥10 cm diameter), and CV of tree dbh) between the rainforest, degraded forest and sclerophyll forest types. A NMS ordination was performed (in PCORD 6) to investigate the plot based variation in above ground carbon storage in relation to the other examined attributes of the examined forests. In the NMS ordination analysis we utilized *Sorensen* and *Orthogonal Principal Axis* (rotation).

The significant explanatory variables dictating the carbon storage of plots were determined using a binomial generalized linear model (GLM) with a logit link function, followed by a backwards, stepwise regression comparison. Prior to creating the global model and candidate model comparisons we performed data exploration and checked for (and removed) correlated predictor variables following the protocol of Zuur, *et al*.^80^. We selected *a priori* a global model in which the carbon storage of plots was a function of: elevation (m), slope (degree), number of fallen logs (≥10 cm diameter), canopy cover (%), mean annual rainfall (mm), tree diameter breast height (cm), tree abundance and forest type (rainforest, degraded forest or sclerophyll). The best model was then determined through a backwards stepwise model comparison whereby nested models were compared using the drop1 function and AIC model values, and the best model was that which contained only significant variables and the lowest AIC model value.

## Additional Information

**How to cite this article**: Alamgir, M. *et al*. Degraded tropical rain forests possess valuable carbon storage opportunities in a complex, forested landscape. *Sci. Rep*. **6**, 30012; doi: 10.1038/srep30012 (2016).

## Figures and Tables

**Table 1:** Generalized linear model result for describing carbon storage (Mg ha^−1^) of the examined forests.

	Estimate	SE	Z value	P(>|z|)
Intercept	1.073	0.1785	6.007	<0.001^
Fallen logs (≥10 cm diameter) (log 10 transformed)	0.2513	0.0779	3.223	0.001#
Tree DBH (cm)	0.1307	0.0072	17.939	<0.001^
Tree abundance	0.0012	7.646e-05	1.128	<0.001^

Only the explanatory variables found to be significant according to their drop1 model comparison values are shown. The fallen logs variable was log 10 transformed prior to the analysis.

(*denotes significance where p = <0.05, ^#^p = <0.01 and ^p = <0.001).
